# Long-term treatment with ruxolitinib for patients with myelofibrosis: 5-year update from the randomized, double-blind, placebo-controlled, phase 3 COMFORT-I trial

**DOI:** 10.1186/s13045-017-0417-z

**Published:** 2017-02-22

**Authors:** Srdan Verstovsek, Ruben A. Mesa, Jason Gotlib, Vikas Gupta, John F. DiPersio, John V. Catalano, Michael W. N. Deininger, Carole B. Miller, Richard T. Silver, Moshe Talpaz, Elliott F. Winton, Jimmie H. Harvey, Murat O. Arcasoy, Elizabeth O. Hexner, Roger M. Lyons, Ronald Paquette, Azra Raza, Mark Jones, Deanna Kornacki, Kang Sun, Hagop Kantarjian, for the COMFORT-I investigators

**Affiliations:** 1 Division of Cancer Medicine, The University of Texas MD Anderson Cancer Center, 1515 Holcombe Blvd, Unit 418, Houston, TX 77030 USA; 2 Mayo Clinic Cancer Center, 13400 E. Shea Blvd, Scottsdale, AZ 85259 USA; 3 Stanford Cancer Institute, 875 Blake Wilbur Drive, Room 2324, Stanford, CA 94305 USA; 4 Princess Margaret Cancer Center, University of Toronto, 610 University Avenue, Suite 5-217, Toronto, M5G 2M9 Canada; 5 Washington University School of Medicine, 660S Euclid Ave., St. Louis, MO 63110 USA; 6 Frankston Hospital and Department of Clinical Haematology, Monash University, 2 Hastings Rd, Frankston, VIC 3199 Australia; 7 University of Utah Huntsman Cancer Institute, 2000 Circle of Hope, Salt Lake City, UT 84112 USA; 8 Saint Agnes Cancer Institute, 900S Caton Ave., Baltimore, MD 21229 USA; 9 Weill Cornell Medical Center, 525 East 68th St., Payson Pavilion 3, New York, NY 10021 USA; 10 University of Michigan, 1500 E Medical Center Dr., Ann Arbor, MI 48109 USA; 11 Emory University School of Medicine, 1365 C Clifton Rd. Suite 1152, Atlanta, GA 30322 USA; 12 Birmingham Hematology and Oncology, 1024 First St North, Birmingham, AL 35204 USA; 13 Duke University Health System, DUMC 3912, Durham, NC 27710 USA; 14 Abramson Cancer Center at the University of Pennsylvania, 3400 Civic Center Blvd., PCAM 2 West Pavilion, Philadelphia, PA 19104 USA; 15 Texas Oncology and US Oncology Research, 4411 Medical Dr., San Antonio, TX 78229 USA; 16 Cedars-Sinai, 10833 Le Conte Ave., 42-121 CHS, Los Angeles, CA 90024 USA; 17 Columbia University Medical Center, Milstein Hospital Building, 6N-435, 177 Fort Washington Ave., New York, NY 10032 USA; 18 Incyte Corporation, 1801 Augustine Cut-Off, Wilmington, DE 19803 USA; 19 Division of Hematology and Hematologic Malignancies and Huntsman Cancer Institute, University of Utah, Salt Lake City, UT 84112 USA

**Keywords:** JAK, Janus kinase, Myelofibrosis

## Abstract

**Background:**

The randomized, double-blind, placebo-controlled, phase 3 COMFORT-I trial evaluated the JAK1/JAK2 inhibitor ruxolitinib in patients with intermediate-2/high-risk myelofibrosis. The primary and planned 3-year analyses of COMFORT-I data demonstrated that ruxolitinib—the first myelofibrosis-approved therapy—reduced splenomegaly and prolonged overall survival versus placebo. Here, we present the final 5-year results.

**Methods:**

Patients managed in Australia, Canada, and the USA were randomized centrally (interactive voice response system) 1:1 to oral ruxolitinib twice daily (15 or 20 mg per baseline platelet counts) or placebo. Investigators and patients were blinded to treatment. The secondary endpoints evaluated in this analysis were durability of a ≥35% reduction from baseline in spleen volume (spleen response) and overall survival, evaluated in the intent-to-treat population. Safety was evaluated in patients who received study treatment.

**Results:**

Patients were randomized (September 2009–April 2010) to ruxolitinib (*n* = 155) or placebo (*n* = 154). At termination, 27.7% of ruxolitinib-randomized patients and 25.2% (28/111) who crossed over from placebo were on treatment; no patients remained on placebo. Patients randomized to ruxolitinib had a median spleen response duration of 168.3 weeks and prolonged median overall survival versus placebo (ruxolitinib group, not reached; placebo group, 200 weeks; HR, 0.69; 95% CI, 0.50–0.96; *P* = 0.025) despite the crossover to ruxolitinib. The ruxolitinib safety profile remained consistent with previous analyses. The most common new-onset all-grade nonhematologic adverse events starting <12 versus ≥48 months after ruxolitinib initiation were fatigue (29.0 vs 33.3%) and diarrhea (27.8 vs 14.6%). New-onset grade 3 or 4 anemia and thrombocytopenia both primarily occurred within the first 6 months, with no cases after 42 months. The most common treatment-emergent adverse event-related deaths in the ruxolitinib-randomized group were sepsis (2.6%), disease progression (1.9%), and pneumonia (1.9%).

**Conclusion:**

The final COMFORT-I results continue to support ruxolitinib as an effective treatment for patients with intermediate-2/high-risk MF.

**Trial registration:**

ClinicalTrials.gov, NCT00952289

## Background

Myelofibrosis (MF) is a Philadelphia chromosome-negative myeloproliferative neoplasm [1] that is often associated with splenomegaly, anemia, and burdensome symptoms that negatively affect quality of life [2, 3]. In addition, patients with MF have shortened survival compared with age- and sex-matched members of the general population [4]. Allogeneic stem cell transplantation is the only potentially curative treatment option [5]. However, transplant-related morbidity and mortality are considerable, and many patients with MF are ineligible because of their age or comorbidities.

Many patients with MF have mutations associated with dysregulation of the Janus kinase (JAK)/signal transducer and activator of transcription pathway. The most common mutations are in *JAK2* (55–65%), *CALR* (15–25%), and *MPL* (5–15%); a relatively small subset of patients is triple negative for mutations in all three genes (10–20%) [6–9].

Ruxolitinib is a JAK1/JAK2 inhibitor approved by the US Food and Drug Administration for patients with intermediate- or high-risk MF, including primary MF (PMF), post-polycythemia vera MF (PPV-MF), and post-essential thrombocythemia MF (PET-MF), as well as patients with PV who have had an inadequate response to or are intolerant of hydroxyurea [10]. Ruxolitinib is also approved by the European Medicines Agency for the treatment of disease-related splenomegaly or symptoms in adult patients with PMF, PPV-MF, or PET-MF and for the treatment of adult patients with PV who are resistant to or intolerant of hydroxyurea [11]. Approval for MF was based on two randomized phase 3 clinical trials in patients with intermediate-2 or high-risk PMF, PPV-MF, or PET-MF [12, 13]. Controlled Myelofibrosis Study with Oral JAK Inhibitor Treatment (COMFORT)-I was a double-blind, placebo-controlled trial, and COMFORT-II was an open-label trial comparing ruxolitinib with the best available therapy. In both trials, ruxolitinib was superior to control interventions, reducing spleen size and improving MF-related symptoms and quality-of-life (QoL) measures. Spleen volume reductions and improvements in measures of QoL at week 24 in COMFORT-I were observed regardless of MF subtype, age, International Prognostic Scoring System (IPSS) risk score, Eastern Cooperative Oncology Group (ECOG) performance status, and baseline hemoglobin level, platelet count, spleen size, and *JAK2*V617F mutation status [14].

Long-term follow-up analyses of the COMFORT studies have indicated durable clinical benefit and are suggestive of a survival advantage with ruxolitinib treatment [15–17]. Most nonhematologic adverse events in COMFORT-I and COMFORT-II were grade 1 or 2, with the rate generally decreasing with long-term ruxolitinib treatment [15, 16]. Dose-dependent cytopenias were the most common hematologic adverse events. These occurred primarily within the first 12 weeks of ruxolitinib treatment and stabilized thereafter in patients continuing therapy, with hemoglobin levels returning to near-baseline levels [15, 16].

This analysis reports the final long-term efficacy and safety results of COMFORT-I after 5 years of ruxolitinib treatment.

## Methods

### Study design and patients

The detailed study design and protocol of the randomized, double-blind, placebo-controlled, phase 3 COMFORT-I trial have been reported previously [12]. The study was conducted in 89 sites across Australia, Canada, and the USA. Briefly, patients with intermediate-2 or high-risk MF and splenomegaly of >5 cm below the left costal margin by palpation were eligible.

The protocol was reviewed and approved by each participating site’s institutional review board. All patients provided written informed consent.

### Randomization and masking

Patients were randomized 1:1 to ruxolitinib or matching placebo tablets by a centralized interactive voice response system (IVRS). Study investigators and patients were blinded to the treatment. Study treatments were provided in encoded bottles, and patient study drug assignments were provided to site staff by the IVRS.

### Procedures

Study treatments, administered orally twice daily, were ruxolitinib (Incyte Corporation, Wilmington, DE; dosing based on baseline platelet counts: 100–200 × 10^9^/L, 15 mg; >200 × 10^9^/L, 20 mg) or placebo. Dose modification was allowed for efficacy and safety. Crossover from placebo to ruxolitinib was permitted before week 24 for protocol-defined worsening splenomegaly. After week 24, patients with protocol-defined worsening symptomatic spleen growth either received unblinded ruxolitinib or discontinued the study; patients with protocol-defined asymptomatic spleen growth were given the option to unblind, after which they were required to receive ruxolitinib or discontinue the study.

This final analysis occurred when all the patients reached the 5-year visit or discontinued participation. Changes from baseline or crossover baseline in spleen volume were assessed by magnetic resonance imaging or computed tomography every 12 weeks from weeks 12 to 72 and every 24 weeks thereafter. Patients who had a spleen volume measurement at baseline and each time point of interest were evaluable to determine if a ≥35% reduction from baseline in spleen volume was achieved; all patients who withdrew before the time point were considered nonresponders.

### Outcomes

The primary endpoint was the proportion of patients who achieved spleen response (≥35% reduction from baseline in spleen volume) at week 24. Secondary endpoints reported in this analysis included duration of spleen response and overall survival (OS).

Hematologic adverse events were based on laboratory abnormalities. Because the majority of the anemia and thrombocytopenia events occurred early in the study, the incidence of new-onset or worsening grade 3 or 4 anemia or thrombocytopenia was assessed at 6-month intervals in patients originally randomized to ruxolitinib. The placebo group was included in only the first 6-month interval because all the patients receiving placebo discontinued or crossed over to ruxolitinib within 3 months of the primary analysis. Nonhematologic adverse events were assessed per National Cancer Institute Common Terminology Criteria for Adverse Events [18]. The incidence of nonhematologic adverse events was assessed in yearly intervals for patients originally randomized to ruxolitinib.

### Statistical analysis

Changes from baseline or crossover baseline in spleen volume were summarized with descriptive statistics. Durability of spleen response and OS were calculated with the Kaplan-Meier method in the intent-to-treat population. OS was calculated based on randomized treatment. Spleen response was considered lost at the first measurement that was no longer a ≥35% reduction from baseline and was also a >25% increase from the nadir. Hazard ratio with 95% confidence interval and *P* values were calculated with the Cox proportional hazards model and the log-rank test. A subgroup analysis of OS was conducted in patients with intermediate-2 or high-risk MF per IPSS criteria [19].

Safety analyses were conducted in all patients who received ≥1 dose of study treatment. The incidence of new-onset or worsening grade ≥3 anemia and thrombocytopenia (based on laboratory data) and of new-onset or worsening all-grade and grade ≥3 nonhematologic adverse events was calculated using the life-table method. The time to the first event censored at the date of the last laboratory evaluation was used for anemia and thrombocytopenia; the earlier discontinuation or date of data cutoff was used for nonhematologic adverse events. Per the life-table method, the incidence of each adverse event was based on the effective sample size of the time interval, which was the number of patients at risk at the beginning of the interval minus half of the censored patients during the time interval.

Statistical analyses were conducted using SAS version 9.2 (SAS Institute, Cary, NC).

The trial was overseen by a data monitoring committee and is registered at ClinicalTrials.gov (NCT00952289).

### Role of the funding source

Conduct of this study and editorial assistance were funded by Incyte Corporation. Incyte Corporation employees worked with external investigators in designing the study, analyzing data, and confirming accuracy of this report. The authors had full access to all the data in the study and had final responsibility for the decision to submit.

## Results

### Patient disposition

**Fig. 1:**
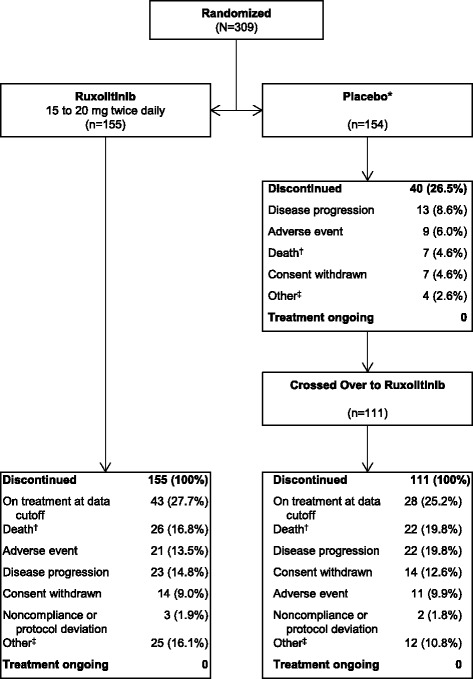
Patient disposition. *Three patients in the placebo group were not evaluable for safety (*n* = 151); these patients were excluded from the calculation of the percentage of patients who discontinued. (*dagger*) Limited to patients whose study discontinuation dates matched their dates of death. (*double dagger*) Including but not limited to the following: received a different therapy, transitioned to commercial ruxolitinib, and loss of response

Patients were recruited between September 2009 and April 2010 and randomized to ruxolitinib (*n* = 155) or placebo (*n* = 154; Fig. 1). All patients were included in the intent-to-treat population; three patients in the placebo group were not evaluable for safety. By the time of the 3-year analysis, all evaluable patients in the placebo group had discontinued (40/151 [26.5%]) or crossed over to ruxolitinib (111/151 [73.5%]) [15]. The median (range) time to crossover was 39.9 (5.0–65.3) weeks. At study termination (i.e., the 5-year data cutoff), 27.7% (43/155) of patients originally randomized to ruxolitinib and 25.2% (28/111) of those who crossed over to ruxolitinib were receiving treatment in the study. An additional four patients in the ruxolitinib-randomized group who discontinued the study transitioned to commercial ruxolitinib.

### Efficacy

#### Spleen response

**Fig. 2:**
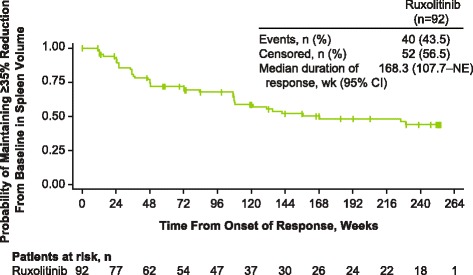
Duration of ≥35% reduction from baseline in spleen volume. Duration of spleen response was evaluated for the 92 patients in the ruxolitinib group who achieved a ≥35% reduction from baseline in spleen volume. NE, not evaluable

**Fig. 3:**
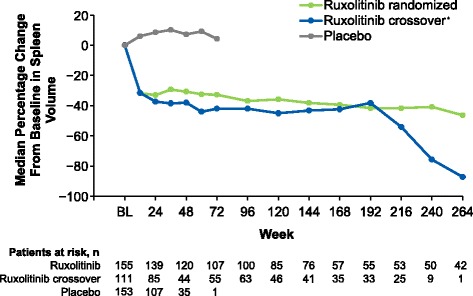
Median percentage change from baseline in spleen volume over time. *For patients in the ruxolitinib crossover group, baseline represents the date of crossover to ruxolitinib. BL, baseline

Among patients originally randomized to ruxolitinib, 59.4% (92/155) had achieved a ≥35% reduction in spleen volume at any time during the study, with a median duration of response of 168.3 weeks (Fig. 2). The proportion of evaluable patients (i.e., those with measurements at baseline and each time point) in the ruxolitinib-randomized group who had a ≥35% reduction from baseline in spleen volume (including patients who had withdrawn as nonresponders) was 41.9% (65/155) at week 24, 36.6% (52/142) at week 48, 34.9% (52/149) at week 96, 28.5% (41/144) at week 144, 22.6% (33/146) at week 192, 20.1% (30/149) at week 240, and 18.5% (27/146) at week 264. Among patients continuing treatment with ruxolitinib, median percentage reductions from baseline in spleen volume were rapid and durable. In the ruxolitinib-randomized group, the median (range) percentage changes from baseline were −33.0% (−75.9 to 25.1%) and −40.8% (−95.9 to 73.3%) at 24 and 240 weeks, respectively; the median (range) percentage changes from crossover baseline were −37.3% (−64.8 to 26.0%) and −75.7% (−85.8 to 49.1%) in the ruxolitinib crossover group (Fig. 3), although the number of evaluable patients was limited at 240 weeks (*n* = 9).

#### Overall survival

**Fig. 4:**
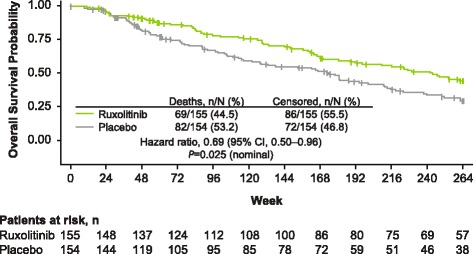
Overall survival. The overall survival analysis included all patients who died during the study or during long-term follow-up after discontinuation of study treatment. HR, hazard ratio

**Fig. 5:**
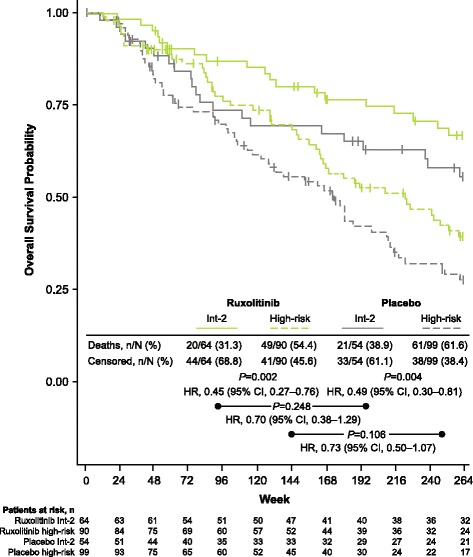
Overall survival by IPSS risk status. In both treatment arms, overall survival was significantly longer for patients with int-2 compared with high-risk MF at diagnosis (ruxolitinib, *P* = 0.002; placebo, *P* = 0.004). Ruxolitinib was associated with nonsignificant survival advantages compared with placebo for both the int-2 and high-risk patient subgroups. HR, hazard ratio; int-2, intermediate-2; IPSS, International Prognostic Scoring System; MF, myelofibrosis

At the time of the final 5-year analysis, median follow-up time for the OS analysis was 268.4 weeks for the ruxolitinib group and 269.0 weeks for the placebo group. Patients randomized to ruxolitinib experienced prolonged OS compared with those in the placebo group. Median OS was not reached in the ruxolitinib-randomized group. Among patients randomized to placebo, median OS was 108 weeks for patients censored at crossover and 200 weeks for all patients (HR, 0.69; 95% CI, 0.50–0.96; *P* = 0.025; Fig. 4). There were a total of 69 deaths (regardless of cause) in the ruxolitinib-randomized group and 82 deaths among those randomized to placebo. In a subgroup analysis by IPSS risk status, there was a nonsignificant trend toward longer OS among patients in the ruxolitinib group compared with the placebo group for both intermediate-2 and high-risk patients (Fig. 5).

### Safety

The median (range) ruxolitinib exposure duration was 149.3 (4.3–296.0) weeks in the ruxolitinib-randomized group and 111.0 (0.9–256.1) weeks in the ruxolitinib crossover group. The median (range) duration of exposure to placebo was 37.1 (3.6–65.3) weeks. Among patients who remained on treatment until study termination, the ruxolitinib exposure duration was 265.4 (249.9–296.0) weeks for the 43 patients in the group randomized to ruxolitinib and 229.6 (200.1–256.1) weeks for the 28 patients in the ruxolitinib crossover group. Ruxolitinib exposure was more than 1 to 2 years for 23/155 (14.8%) and 15/111 (13.5%) patients in the ruxolitinib-randomized group and the ruxolitinib crossover group, respectively; more than 2 to 3 years for 22/155 (14.2%) and 16/111 (14.4%) patients; more than 3 to 4 years for 19/155 (12.3%) and 11/111 (9.9%) patients; and more than 4 years for 62/155 (40.0%) and 35/111 (31.5%) patients.

The incidence of new-onset nonhematologic adverse events generally stabilized or decreased with long-term treatment in the ruxolitinib-randomized group (Tables 1 and 2). The most common new-onset all-grade nonhematologic adverse events starting ≥48 months after ruxolitinib initiation were fatigue (33.3%), pneumonia (16.4%), constipation (16.0%), cough (15.4%), and headache (15.4%); the most common grade 3 or 4 adverse events were pneumonia (15.6%), congestive cardiac failure (6.2%), sepsis (6.2%), and squamous cell carcinoma (6.2%).

**Table 1:** Incidence of new-onset all-grade nonhematologic adverse events in the ruxolitinib group regardless of causality, grouped by treatment time interval

	Ruxolitinib (*n* = 155)				
	0– < 12 Months	12– < 24 Months	24– < 36 Months	36– < 48 Months	≥48 Months
Event,*n/N (%)					
Fatigue	43/148.5 (29.0)	14/92.0 (15.2)	10/65.5 (15.3)	5/45.0 (11.1)	7/21.0 (33.3)
Diarrhea	41/147.5 (27.8)	6/89.0 (6.7)	7/65.0 (10.8)	5/46.5 (10.8)	3/20.5 (14.6)
Ecchymosis	31/146.0 (21.2)	10/96.0 (10.4)	4/70.0 (5.7)	1/55.0 (1.8)	1/25.0 (4.0)
Dyspnea	28/146.0 (19.2)	10/98.5 (10.2)	2/70.0 (2.9)	2/54.5 (3.7)	3/25.0 (12.0)
Dizziness	26/144.0 (18.1)	10/96.0 (10.4)	2/66.5 (3.0)	1/49.5 (2.0)	1/21.5 (4.7)
Pain in extremity	26/144.5 (18.0)	6/97.0 (6.2)	3/71.0 (4.2)	2/51.5 (3.9)	1/21.5 (4.7)
Peripheral edema	26/145.5 (17.9)	7/99.5 (7.0)	8/75.0 (10.7)	3/53.5 (5.6)	2/23.0 (8.7)
Headache	24/144.5 (16.6)	5/99.0 (5.1)	3/75.0 (4.0)	4/58.0 (6.9)	4/26.0 (15.4)
Nausea	24/144.5 (16.6)	7/102.5 (6.8)	4/79.0 (5.1)	5/61.0 (8.2)	4/27.5 (14.5)
Constipation	21/145.0 (14.5)	10/105.0 (9.5)	8/78.5 (10.2)	4/56.5 (7.1)	4/25.0 (16.0)
Abdominal pain	20/144.5 (13.8)	6/106.0 (5.7)	3/84.0 (3.6)	4/66.0 (6.1)	4/29.5 (13.6)
Insomnia	20/144.5 (13.8)	7/104.5 (6.7)	3/80.0 (3.8)	1/62.5 (1.6)	1/28.0 (3.6)
Vomiting	20/145.5 (13.7)	3/105.5 (2.8)	2/82.5 (2.4)	4/64.5 (6.2)	4/29.0 (13.8)
Pyrexia	20/148.0 (13.5)	8/109.5 (7.3)	7/82.5 (8.5)	3/62.0 (4.8)	2/27.5 (7.3)
Cough	19/145.0 (13.1)	14/105.5 (13.3)	3/74.5 (4.0)	4/58.5 (6.8)	4/26.0 (15.4)
Arthralgia	17/144.0 (11.8)	6/103.0 (5.8)	6/75.5 (7.9)	6/53.5 (11.2)	3/21.5 (14.0)
Muscle spasms	14/143.0 (9.8)	3/105.0 (2.9)	7/81.0 (8.6)	6/58.0 (10.3)	1/23.0 (4.3)
Back pain	13/143.0 (9.1)	11/106.5 (10.3)	0	4/58.0 (6.9)	3/25.5 (11.8)
Night sweats	13/143.0 (9.1)	3/105.5 (2.8)	3/81.5 (3.7)	1/61.5 (1.6)	4/28.0 (14.3)
Pneumonia	13/145.0 (9.0)	7/110.0 (6.4)	3/82.5 (3.6)	3/65.0 (4.6)	5/30.5 (16.4)
Upper respiratory tract infection	11/143.0 (7.7)	12/108.0 (11.1)	4/74.5 (5.4)	4/55.0 (7.3)	3/24.0 (12.5)
Fall	7/143.5 (4.9)	2/111.5 (1.8)	1/87.0 (1.1)	3/68.5 (4.4)	4/30.5 (13.1)
Musculoskeletal pain	7/143.0 (4.9)	5/112.0 (4.5)	7/85.5 (8.2)	2/62.5 (3.2)	4/29.0 (13.8)
Pruritus	7/142.5 (4.9)	8/110.5 (7.2)	1/81.0 (1.2)	1/63.5 (1.6)	3/29.0 (10.3)
Herpes zoster	3/143.5 (2.1)	4/115.5 (3.5)	3/87.5 (3.4)	3/66.0 (4.5)	3/29.0 (10.3)
Squamous cell carcinoma	0	1/116.5 (0.9)	2/91.5 (2.2)	2/70.5 (2.8)	4/32.0 (12.5)

*Occurring in >10% of patients in the ruxolitinib group in ≥1 yearly interval

**Table 2:** Incidence of new-onset grade 3 or 4 nonhematologic adverse events in the ruxolitinib group regardless of causality, grouped by treatment time interval

	Ruxolitinib (*n* = 155)				
	0– < 12 Months	12– < 24 Months	24– < 36 Months	36– < 48 Months	≥48 Months
Event,*n/N (%)					
Fatigue	9/144.5 (6.2)	1/113.0 (0.9)	3/90.0 (3.3)	1/69.5 (1.4)	0
Pneumonia	8/144.0 (5.6)	4/112.0 (3.6)	3/86.0 (3.5)	2/67.5 (3.0)	5/32.0 (15.6)
Abdominal pain	6/143.5 (4.2)	0	3/93.5 (3.2)	1/72.5 (1.4)	1/32.0 (3.1)
Arthralgia	3/142.5 (2.1)	0	0	1/70.0 (1.4)	0
Diarrhea	3/143.5 (2.1)	0	0	1/72.5 (1.4)	0
Dyspnea	3/143.5 (2.1)	1/116.5 (0.9)	2/92.5 (2.2)	1/71.5 (1.4)	1/31.5 (3.2)
Pain in extremity	3/142.5 (2.1)	0	1/89.5 (1.1)	1/69.5 (1.4)	1/30.5 (3.3)
Acute myeloid leukemia	2/143.5 (1.4)	0	1/93.0 (1.1)	2/74.0 (2.7)	0
Fall	2/142.5 (1.4)	1/114.5 (0.9)	0	2/71.0 (2.8)	1/30.5 (3.3)
Gastrointestinal hemorrhage	2/142.5 (1.4)	1/115.0 (0.9)	0	0	0
Hyperuricemia	2/142.5 (1.4)	1/114.5 (0.9)	0	1/71.5 (1.4)	0
Hypoxia	2/142.5 (1.4)	0	2/92.0 (2.2)	0	1/31.5 (3.2)
Muscular weakness	2/143.0 (1.4)	0	1/91.5 (1.1)	0	0
Septic shock	2/143.5 (1.4)	0	0	0	0
Acute renal failure	1/142.5 (0.7)	1/116.0 (0.9)	3/93.0 (3.2)	2/72.5 (2.8)	1/31.5 (3.2)
Back pain	1/142.5 (0.7)	2/116.0 (1.7)	0	0	0
Congestive cardiac failure	1/142.5 (0.7)	0	1/92.0 (1.1)	0	2/32.5 (6.2)
Epistaxis	1/143.0 (0.7)	2/117.0 (1.7)	0	0	0
Sepsis	1/143.0 (0.7)	2/116.5 (1.7)	2/92.5 (2.2)	1/73.0 (1.4)	2/32.5 (6.2)
Upper abdominal pain	1/143.0 (0.7)	0	2/92.5 (2.2)	0	0
Cellulitis	0	0	0	2/73.5 (2.7)	0
Myocardial infarction	0	1/117.0 (0.9)	0	2/73.5 (2.7)	0
Osteoarthritis	0	0	1/92.5 (1.1)	0	2/32.5 (6.2)
Osteomyelitis	0	0	0	2/73.0 (2.7)	0
Squamous cell carcinoma	0	1/116.5 (0.9)	0	0	2/32.5 (6.2)
Urinary tract infection	0	1/116.5 (0.9)	1/92.0 (1.1)	0	2/33.0 (6.1)
Wound infection	0	0	0	0	2/33.0 (6.1)

*Occurring in ≥2 patients in the ruxolitinib group in any yearly interval

**Fig. 6:**
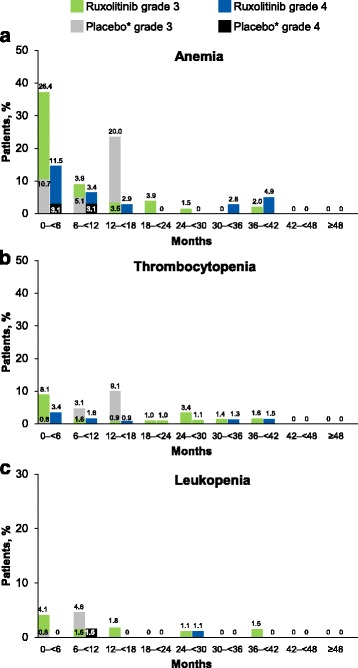
Incidence of new or worsening grade 3 or 4 **a** anemia, **b** thrombocytopenia, and **c** leukopenia over time. Anemia, thrombocytopenia, and leukopenia were based on hematologic laboratory abnormalities. (*asterisk*) Placebo arm data are only shown up to 6 months because all patients randomized to placebo crossed over or discontinued within 3 months of the primary analysis

**Fig. 7:**
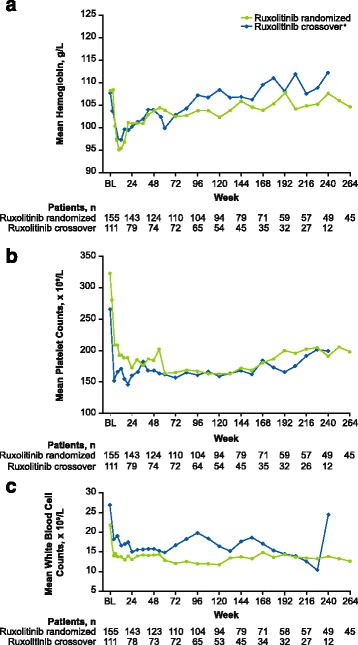
Mean blood counts over time in the ruxolitinib randomized and ruxolitinib crossover groups. Blood counts were based on measurements of **a** hemoglobin level, **b** platelet counts, and **c** white blood cell counts. *For patients in the ruxolitinib crossover group, BL represents the date of crossover to ruxolitinib. BL, baseline

Anemia and thrombocytopenia (per abnormal hematologic laboratory values) occurred in most patients in the ruxolitinib-randomized group (98.7 and 83.9%, respectively). The rates of new or worsening grade 3 or 4 anemia, thrombocytopenia, and leukopenia were the highest within the first 6 months of treatment, decreasing thereafter (Fig. 6). No patients in the ruxolitinib-randomized group had new or worsening grade 3 or 4 anemia, thrombocytopenia, or leukopenia after month 42. In agreement with the hematologic laboratory abnormalities over time in the ruxolitinib-randomized group, mean hemoglobin levels decreased during the first 12 weeks of treatment with randomized or crossover ruxolitinib but increased toward baseline levels and stabilized thereafter (Fig. 7). Similarly, mean platelet and white blood cell counts also decreased during the first 12 weeks of treatment with ruxolitinib, after which they remained stable (Fig. 7). In agreement with these blood count dynamics, the mean (SD) number of transfusions per month in the ruxolitinib group peaked between weeks 4 and 8 (1.2 [1.75]) then decreased steadily until weeks 36 to 48 (0.7 [1.35]), stabilizing thereafter.

Serious adverse events occurred at any time during treatment with ruxolitinib in 103/155 (66.5%) patients in the ruxolitinib-randomized group and 74/111 (66.7%) patients in the ruxolitinib crossover group. The most frequent serious adverse events, occurring in ≥4% of patients in the ruxolitinib-randomized or crossover groups, were pneumonia (randomized, 15.5%; crossover, 10.8%), anemia (11.0%; 11.7%), sepsis (4.5%; 3.6%), and congestive cardiac failure (3.2%; 4.5%).

Throughout the course of the study, adverse events resulted in a ruxolitinib dose decrease in 88/155 (56.8%) patients in the ruxolitinib-randomized group and 45/111 (40.5%) patients in the ruxolitinib crossover group. Thrombocytopenia was the most frequent cause of dose decreases, occurring in 75/155 (48.4%) and 36/111 (32.4%) patients in the ruxolitinib-randomized and crossover groups, respectively.

Adverse events resulted in discontinuation of treatment in 50/155 (32.3%) patients originally randomized to ruxolitinib, 39/111 (35.1%) in the ruxolitinib crossover group, and 19/151 (12.6%) during treatment with placebo. The most common reasons, occurring in ≥2.0% of patients treated with ruxolitinib, were disease progression (3.2%), acute myeloid leukemia (AML; 2.6%), and anemia (2.6%) in the ruxolitinib-randomized group, and thrombocytopenia (3.6%) and AML (3.6%) in the ruxolitinib crossover group.

Herpes zoster infections occurred at higher rates in patients treated with ruxolitinib compared with placebo (Table 3). All herpes zoster events in the ruxolitinib-randomized group were grade 1 or 2, occurring in three or four patients each year. Among all patients treated with ruxolitinib, the majority of cases were single episodes that were grade 2 or lower and resolved without long-term sequelae. The only serious event of herpes zoster infection occurred in a patient randomized to placebo after crossing over to ruxolitinib.

**Table 3:** Exposure-adjusted rates of select adverse events

Median duration of exposure, d	Ruxolitinib randomized (*n* = 155)		Ruxolitinib crossover (*n* = 111)*		During placebo treatment (*n* = 151)*	
	1045.0		777.0		260.0	
	All grade	Grade 3 or 4	All grade	Grade 3 or 4	All grade	Grade 3 or 4
Event, n/PYE (rate per 100 PYE)						
Infections and infestations^a^						
Upper respiratory tract infection	34/398.0 (8.5)	0	22/230.5 (9.5)	0	15/96.9 (15.5)	1/96.9 (1.0)
Urinary tract infection	31/414.3 (7.5)	4/414.3 (1.0)	16/240.5 (6.7)	3/240.5 (1.2)	7/101.3 (6.9)	1/101.3 (1.0)
Pneumonia	31/432.3 (7.2)	22/432.3 (5.1)	18/253.6 (7.1)	8/253.6 (3.2)	11/102.4 (10.7)	8/102.4 (7.8)
Herpes zoster	16/452.5 (3.5)	0	14/241.2 (5.8)	1/242.2 (0.4)	1/104.1 (1.0)	0
Bronchitis	14/450.5 (3.1)	0	11/244.9 (4.5)	3/244.9 (1.2)	2/104.2 (1.9)	0
Nasopharyngitis	14/449.1 (3.1)	0	9/253.6 (3.5)	0	9/98.4 (9.1)	0
Sinusitis	12/453.2 (2.6)	1/453.2 (0.2)	7/252.3 (2.8)	0	3/102.7 (2.9)	1/102.7 (1.0)
Cellulitis	10/467.8 (2.1)	2/467.8 (0.4)	3/262.6 (1.1)	0	2/103.5 (1.9)	0
Influenza	8/469.0 (1.7)	0	3/266.0 (1.1)	1/266.0 (0.4)	0	0
Sepsis	8/480.3 (1.7)	8/480.3 (1.7)	4/267.4 (1.5)	4/267.4 (1.5)	2/104.0 (1.9)	1/104.0 (1.0)
Tooth abscess	7/476.3 (1.5)	1/476.3 (0.2)	4/261.3 (1.5)	0	0	0
Oral herpes	6/469.8 (1.3)	0	2/269.1 (0.7)	0	2/103.2 (1.9)	0
Skin infection	5/469.8 (1.1)	0	3/269.5 (1.1)	0	1/104.4 (1.0)	0
Viral infection	5/471.2 (1.1)	0	2/265.4 (0.8)	0	0	0
Viral gastroenteritis	4/470.6 (0.9)	0	1/270.1 (0.4)	0	2/103.5 (1.9)	0
Diverticulitis	4/475.0 (0.8)	1/475.0 (0.2)	3/268.5 (1.1)	1/268.5 (0.4)	2/103.6 (1.9)	0
Ear infection	4/473.3 (0.8)	0	4/267.8 (1.5)	0	0	0
Fungal infection	4/479.4 (0.8)	0	2/267.6 (0.7)	1/267.6 (0.4)	2/103.8 (1.9)	0
Localized infection	4/479.1 (0.8)	0	1/269.2 (0.4)	1/269.2 (0.4)	1/104.1 (1.0)	0
Lower respiratory tract infection	4/476.9 (0.8)	0	1/270.5 (0.4)	0	2/103.3 (1.9)	1/103.3 (1.0)
Septic shock	2/484.6 (0.4)	2/484.6 (0.4)	3/270.5 (1.1)	3/270.5 (1.1)	0	0
Neoplasms						
Basal cell carcinoma	12/450.9 (2.7)	2/450.9 (0.4)	10/252.7 (4.0)	2/252.7 (0.8)	4/103.7 (3.9)	0
Squamous cell carcinoma	10/462.6 (2.2)	2/462.6 (0.8)	10/252.0 (4.0)	3/252.0 (1.2)	4/102.9 (3.9)	0
Squamous cell carcinoma of the skin	9/470.2 (1.9)	3/470.2 (0.6)	3/266.4 (1.1)	1/266.4 (0.4)	1/104.7 (1.0)	0
Acute myeloid leukemia	5/483.8 (1.0)	5/483.8 (1.0)	5/270.1 (1.9)	5/270.1 (1.9)	0	0

*PYE*, patient-years of exposure

*Adverse events that occurred following the first dose of ruxolitinib (ie, after crossover from placebo) were included in the ruxolitinib crossover group

^a^Occurring in ≥5 patients treated with ruxolitinib

Sepsis occurred at similar rates between patients treated with ruxolitinib and those receiving placebo (Table 3). All sepsis events were grade 3 or 4, with the exception of one grade 2 event in the placebo group. Serious events of sepsis and septic shock occurred at rates of 1.5 and 0.4 per 100 patient-years of exposure in the ruxolitinib-randomized group and 1.5 each in the ruxolitinib crossover group.

Nonmelanoma skin cancers, including basal cell carcinoma and squamous cell carcinoma of the skin, occurred at similar rates between patients treated with ruxolitinib and those receiving placebo (Table 3). Basal cell carcinoma occurred at a rate of 2.7 per 100 patient-years of exposure in the ruxolitinib-randomized group, 4.0 in the ruxolitinib crossover group, and 3.9 among patients during treatment with placebo (Table 3). There were two cases of basal cell carcinoma in the ruxolitinib crossover group; in both cases, patients had a history of skin cancer.

Disease transformation to AML occurred in five patients each in the ruxolitinib-randomized and ruxolitinib crossover groups; no patients developed AML during treatment with placebo (Table 3). Overall, AML occurred in five male and five female patients. The median (range) time from the first ruxolitinib dose to AML diagnosis was 838 (157–1150) days in the ruxolitinib-randomized group and 376 (21–666) days in the ruxolitinib crossover group; median (range) time from MF diagnosis to AML diagnosis was 1190 (699–1708) days and 1015 (372–11,971) days, respectively. Prior medications for the treatment of MF in patients who developed AML were hydroxyurea (ruxolitinib-randomized group, *n* = 2; ruxolitinib crossover group, *n* = 2) and lenalidomide, and corticosteroids (all in one patient in the ruxolitinib crossover group); three patients in the ruxolitinib-randomized group and two in the ruxolitinib crossover group had no prior treatments for MF.

Overall, 28/155 (18.1%) patients in the ruxolitinib-randomized group and 39/151 (25.8%) in the placebo randomized group experienced a treatment-emergent adverse event that resulted in death while on study or within 28 days of the last dose of study drug. Among the patients randomized to placebo, a treatment-emergent adverse event led to death in 11/151 (7.3%) patients during treatment with placebo and 28/111 (25.2%) patients after crossover to ruxolitinib (Table 4).

**Table 4:** Treatment-emergent adverse events resulting in death*

Cause of death, n (%)^a^	Ruxolitinib randomized (*n* = 155)	After ruxolitinib crossover^b^ (*n* = 111)	During placebo treatment (*n* = 151)
Death caused by any treatment-emergent adverse event	28 (18.1)	28 (25.2)	11 (7.3)
Sepsis	4 (2.6)	2 (1.8)	1 (0.7)
Disease progression	3 (1.9)	4 (3.6)	3 (2.0)
Pneumonia	3 (1.9)	1 (0.9)	1 (0.7)
Acute myeloid leukemia	2 (1.3)	3 (2.7)	0
Cerebral hemorrhage	2 (1.3)	1 (0.9)	1 (0.7)
Septic shock	2 (1.3)	2 (1.8)	0
Acute renal failure	1 (0.6)	1 (0.9)	0
Anemia	1 (0.6)	0	0
Cardiac arrest	1 (0.6)	0	0
Death, unspecified	1 (0.6)	1 (0.9)	0
Falling injury	1 (0.6)	0	0
Hemorrhagic shock	1 (0.6)	1 (0.9)	0
Metastatic NSCLC	1 (0.6)	0	0
Multiorgan failure	1 (0.6)	0	1 (0.7)
Muscular weakness	1 (0.6)	0	0
Myocardial infarction	1 (0.6)	1 (0.9)	0
Pancreatic carcinoma	1 (0.6)	0	0
Renal failure	1 (0.6)	0	0
Respiratory failure	1 (0.6)	0	0
Splenic infarction	1 (0.6)	0	0
Congestive cardiac failure	0	2 (1.8)	0
Myelofibrosis	0	2 (1.8)	1 (0.7)
Cardiac failure	0	1 (0.9)	0
Pneumonia aspiration	0	2 (1.8)	0
Anastomotic hemorrhage	0	1 (0.9)	0
Cholecystitis	0	1 (0.9)	0
Delirium	0	1 (0.9)	0
Road traffic accident	0	1 (0.9)	0
Splenic rupture	0	1 (0.9)	0
Suicide	0	1 (0.9)	0
Gastrointestinal hemorrhage	0	0	1 (0.7)
Intestinal perforation	0	0	1 (0.7)
Staphylococcal infection	0	0	1 (0.7)

*NSCLC*, non-small cell lung cancer

*Limited to fatal treatment-emergent adverse events occurring during treatment with study drug or within 28 days of the last dose of study drug

^a^Patient deaths were counted once under each Medical Dictionary for Regulatory Activities system organ class and preferred term, and therefore individual patients may have had >1 cause of death

^b^Fatal treatment-emergent adverse events that occurred following the first dose of ruxolitinib (ie, after crossover from placebo) were included in the ruxolitinib crossover group

## Discussion

This final analysis of the COMFORT-I trial demonstrated that treatment with ruxolitinib was associated with rapid and durable reductions in splenomegaly and significantly longer OS compared with patients originally randomized to placebo. Patient risk of death was approximately 30% lower in the ruxolitinib group compared with placebo, despite the crossover from placebo to ruxolitinib. Given that COMFORT-I was restricted to patients with intermediate-2 or high-risk MF with splenomegaly, OS data suggest that delaying treatment with ruxolitinib may worsen outcomes and that studies evaluating ruxolitinib in patients with earlier MF disease states may be warranted.

The exact mechanism by which ruxolitinib prolongs survival and ameliorates splenomegaly remains unclear, but it is rational to hypothesize that the downstream effects of ruxolitinib confer changes in cytokines, metabolic properties, and *JAK2*V617F allele burden that may play a role. Ruxolitinib has been associated with reductions in inflammatory cytokines and markers of inflammation [12], improvements in measures of metabolic and nutrition status [20], reduced fibrosis in some patients [17], and reductions in *JAK2*V617F allele burden [21]. In COMFORT-I patients receiving long-term treatment with ruxolitinib, relationships have been identified between reductions in spleen volume and (1) increases in body weight and normalization of serum albumin and total cholesterol levels [20] and (2) reductions in *JAK2*V617F allele burden in some patients [21]. In addition, ruxolitinib has been associated with improvements in spleen volume and OS in a wide variety of patient subgroups stratified by MF subtype, age, IPSS risk score, ECOG performance status, and baseline hemoglobin level, platelet count, spleen size, and *JAK2*V617F mutation status [14]. Future work will be required to elucidate the mechanism by which ruxolitinib is efficacious and if there are any related disease markers or patient characteristics that could be helpful in identifying the types of patients who may benefit the most from ruxolitinib treatment.

Overall, the safety profile was supportive of long-term treatment with ruxolitinib, with no unexpected safety signals. The nonhematologic adverse event rates generally remained stable or decreased with prolonged ruxolitinib treatment duration and were consistent with those reported in previous analyses of the COMFORT-I study [12, 15]. As expected, based on the mechanism of action of ruxolitinib as a JAK1/JAK2 inhibitor [22, 23], thrombocytopenia and anemia occurred in most patients treated with ruxolitinib. Anemia and thrombocytopenia can be managed with dose adjustments and, for some patients with anemia, red blood cell transfusions [24]. Indeed, although thrombocytopenia was the most common cause for ruxolitinib dose reduction in COMFORT-I, thrombocytopenia and anemia resulted in relatively few discontinuations (each ≤3.6% in the ruxolitinib-randomized and crossover groups). Mean hemoglobin, platelet, and white blood cell levels stabilized after 12 weeks of treatment, with hemoglobin levels returning to near-baseline levels thereafter; however, this finding must be interpreted taking into account the positive selection of patients remaining on study. Mean blood transfusion rates were in agreement with these trends. Nevertheless, ruxolitinib may provide a survival benefit even in the presence of anemia. In a pooled analysis of COMFORT-I and COMFORT-II 3-year data, treatment with ruxolitinib was associated with a survival advantage regardless of anemia at baseline (3-year OS probability: ruxolitinib, 0.66; control, 0.57) or after initiating study treatment (3-year OS probability: ruxolitinib, 0.87; control, 0.66) [25].

Herpes zoster infections occurred at higher rates among patients treated with ruxolitinib compared with placebo. The incidence of herpes zoster infections increased with longer exposure to ruxolitinib (0–12 months’ exposure, 2.1%; ≥48 months’ exposure, 10.3%). However, all but one case was grade 1 or 2, and it is unclear if this increase was clinically relevant. Other infections, including pneumonia, sepsis, upper respiratory tract infection, and urinary tract infection, occurred at similar or lower rates with ruxolitinib compared with placebo; however, pneumonia was the most common new-onset grade 3 or 4 adverse event observed after 48 months of treatment with ruxolitinib. Nonmelanoma skin cancers were observed in patients treated with ruxolitinib; however, these occurred at rates that were similar to or lower than those observed during treatment with placebo. Finally, the incidence of AML transformation in the ruxolitinib-randomized and crossover groups was consistent with previous reports in patients with MF [26, 27]. Although no patients developed AML during treatment with placebo, the median exposure (37.1 weeks) may not have been long enough to observe AML transformations considering that the median time from the first ruxolitinib dose to AML diagnosis was 119.7 weeks in the ruxolitinib-randomized group.

Overall, 48.9% of the COMFORT-I patient population had died by the time of the final 5-year analysis. Causes of death were generally consistent with expected morbidities resulting from MF progression and/or other underlying disease processes (e.g., infections, transformation to AML), particularly in elderly and chronically ill patients. The most common adverse events leading to death in COMFORT-I were sepsis or septic shock, followed by disease progression, pneumonia, and transformation to AML. Eleven patients treated with ruxolitinib died because of a cardiovascular, thrombotic, or hemorrhagic event. In comparison, an international retrospective analysis of 1131 patients with PMF enrolled between 1980 and 2007 reported that the leading causes of death were transformation to AML, disease progression, thrombosis and cardiovascular complications, and infection [19].

## Conclusions

This final analysis of the COMFORT-I study included 5 years of treatment duration and demonstrated that long-term ruxolitinib treatment in patients with intermediate-2 or high-risk MF was associated with durable reductions in spleen size and significantly longer OS compared with placebo. The safety profile continued to remain consistent with previous COMFORT-I and COMFORT-II analysis [12, 13, 15–17], with no new or unexpected adverse events identified with long-term treatment. Collectively, these data and similar findings in the 5-year analysis of the COMFORT-II study [17] support ruxolitinib as an effective long-term treatment option for patients with intermediate-2 or high-risk MF.

## Abbreviations

AML: Acute myeloid leukemia
BL: Baseline
COMFORT: Controlled Myelofibrosis Study with Oral JAK Inhibitor Treatment
ECOG: Eastern Cooperative Oncology Group
HR: Hazard ratio
IPSS: International Prognostic Scoring System
IVRS: Interactive voice response system
JAK: Janus kinase
MF: Myelofibrosis
NE: Not evaluable
NSCLC: Non-small cell lung cancer
OS: Overall survival
PET-MF: Post-essential thrombocythemia myelofibrosis
PMF: Primary myelofibrosis
PPV-MF: Post-polycythemia vera myelofibrosis
PV: Polycythemia vera
PYE: Patient-years exposure
QoL: Quality of life
